# Septal total atrial conduction time for prediction of atrial fibrillation in embolic stroke of unknown source: a pilot study

**DOI:** 10.1007/s00392-019-01501-2

**Published:** 2019-06-24

**Authors:** Jan-Thorben Sieweke, Saskia Biber, Karin Weissenborn, Peter U. Heuschmann, Muharrem Akin, Florian Zauner, Maria M. Gabriel, Ramona Schuppner, Dominik Berliner, Johann Bauersachs, Gerrit M. Grosse, Udo Bavendiek

**Affiliations:** 1 Department of Cardiology and Angiology, Hannover Medical School, Hannover, Germany; 2 Department of Neurology, Hannover Medical School, Hannover, Germany; 3 Insitute of Clinical Epidemiology and Biometry, University Würzburg, Comprehensive Heart Failure Center, University of Würzburg, Clinical Trial Center, University Hospital Würzburg, Würzburg, Germany

**Keywords:** Stroke, ESUS, Echocardiography, Atrial fibrillation

## Abstract

**Background:**

Subclinical atrial fibrillation (AF) is the underlying cause in a relevant part of patients with embolic stroke of unknown source (ESUS). This pilot study aims to identify novel echocardiographic parameters predicting AF subsequently detected in patients originally hospitalized with ESUS.

**Methods and results:**

Patients with acute ischemic stroke [baseline diagnosis of ESUS (*n* = 69), stroke of macro- or microvascular cause (*n* = 16/25), stroke caused by AF (*n* = 5)] and controls with paroxysmal AF without acute ischemic stroke (n = 22) as well as healthy controls of young and old age (*n* = 21/17) in sinus rhythm were included (overall *n* = 175). Echocardiography was performed in all participants. Prolonged Holter-ECG-monitoring was performed in all stroke patients. In the overall cohort, septal total atrial conduction time (sPA-TDI), left atrial (LA) volume index to tissue Doppler velocity (LAVI/a`) and second negative peak strain rate during LA contraction (SRa), representing echocardiographic parameters of LA remodelling and function, were statistically significant different in patients with and without AF and predictive for subclinical AF (multivariate regression analysis: sPA-TDI: HR 1.06 [1.04–1.08], *p* < 0.001; LAVI/a`: HR 0.85, [0.74–0.97], p = 0.02; SRa: HR 2.35 [0.9–5.5], *p* = 0.05). Multivariate Cox regression analysis revealed sPA-TDI as an independent predictor of AF in ESUS patients (sPA-TDI: HR 1.10 [1.04–1.17], *p* = 0.001). A sPA-TDI of 126 ms strictly discriminated between presence and absence of subclinical AF within 48 h after initiation of Holter-ECG-monitoring in ESUS patients.

**Conclusions:**

sPA-TDI seems to be a strong independent predictor of subclinical AF in patients hospitalized for ESUS and might support risk-stratified clinical decision making in these patients.

**Graphic abstract:**

Septal Total Atrial Conduction Time (sPA-TDI) determined by echocardiography for prediction of Atrial Fibrillation in Embolic Stroke of Unknown Source (ESUS). 
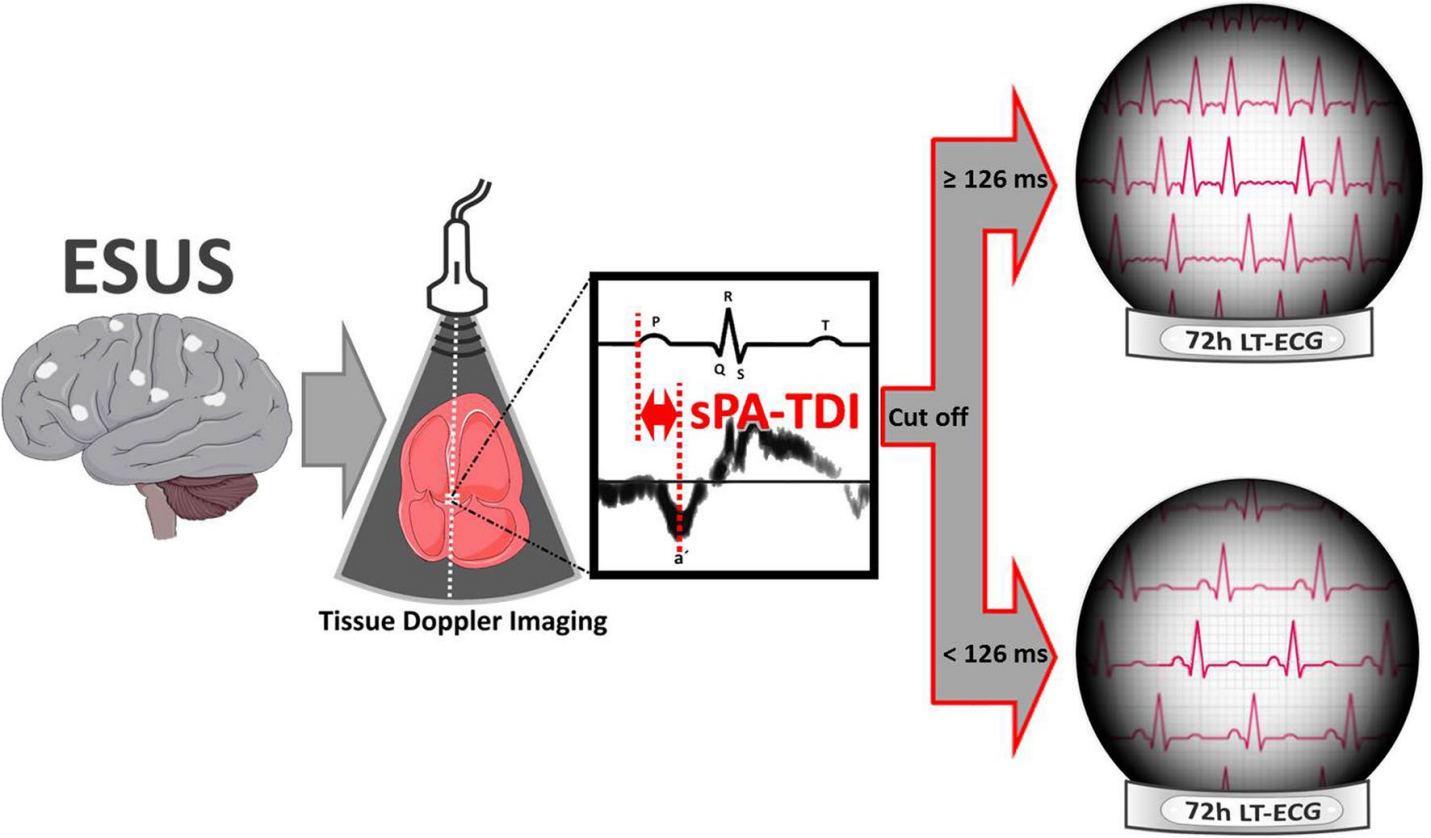

**Electronic supplementary material:**

The online version of this article (10.1007/s00392-019-01501-2) contains supplementary material, which is available to authorized users.

## Introduction

Despite extensive medical work-up in about 25% of stroke patients the underlying cause remains unknown (undefined stroke) [1]. Within a significant part of undefined strokes a more clinically useful entity showing an embolic cerebral imaging pattern has been proposed as Embolic Stroke of Unknown Source (ESUS) [2]. It is postulated that previously non-detected AF is the major underlying cause in a relevant part of ESUS.

Although apparently promising for the detection of AF in cryptogenic stroke, extended cardiac rhythm monitoring requires significant analytical resources and causes patient burden [3–6]. Therefore, powerful predictors of subclinical AF would be important to screen and identify those patients with cryptogenic stroke and especially ESUS, who might benefit from extended cardiac rhythm monitoring or even anticoagulant therapy. In addition, in patients with determined stroke etiology concomitant subclinical AF might exist and significantly impact stroke recurrence due to lack of therapeutic anticoagulation.

Echocardiography is a widely available and important imaging tool to determine left atrial (LA) function and remodeling, especially in the evaluation of patients with AF [7–11]. Parameters of LA strain analyzed by speckle tracking echocardiography reflect LA function and were associated with AF, fibrosis and stroke [8, 9]. In addition, total atrial conduction time (PA-TDI interval) and the ratio of LA volume index to tissue Doppler a` (LAVI/a`) correlate with LA remodeling and fibrosis and serve as a predictive marker for AF [10–14].

The present pilot study investigated whether echocardiographic parameters indicating LA remodeling, fibrosis and LA function predict subclinical AF in patients hospitalized for ESUS compared to patients with other stroke etiology and controls with and without AF.

## Methods

### Study design and participants

The present study is a prospective, single-blinded, single center controlled pilot-study, which was conducted in accordance with the Declaration of Helsinki after approval by the local ethics committee of Hannover Medical School (Application Number: 3316-2016). We consecutively screened patients presenting in sinus rhythm for eligibility, who were admitted to our local certified Stroke Unit, the cardiology ward and a group of volunteers without documented AF. Detailed patient inclusion and exclusion criteria are listed in the supplement.

Included participants were allocated into cohorts (Controls and Stroke) and categorized into groups: (1) Control cohort: yCw/oAF = healthy, young volunteers without documented AF (health care professionals); oCw/oAF = healthy volunteers of old age without documented AF (spouse of stroke patients) and CpAFw/oS = participants with documented chronic paroxysmal AF without acute stroke. Control groups were included for internal validation of echocardiographic parameters indicating LA remodeling [8–11] (2) Stroke cohort: ESUS ± AF = patients with embolic stroke of unknown source ± AF [2], CES-AF = cardio-embolic stroke based on previously diagnosed chronic paroxysmal AF; MavS = stroke due to macro-vascular cause; MivS = stroke due to micro-vascular cause. Classification to stroke groups including ESUS was performed before Holter ECG-Monitoring in respect of results of brain imaging, continuous ECG monitoring with a central monitoring unit starting at stroke unit admission lasting at least 24 h, 12-channel surface ECG recordings, echocardiography and additional examinations if indicated. After classification to stroke groups, Holter-ECG-Monitoring scheduled for 72 h was realized. If atrial fibrillation was recorded within the first 24 h after admission in continuous-ECG-monitoring at the stroke-unit, 12-channel surface ECG and/or during echocardiography, patients were classified to the CES-AF group. Because of the design and purpose of the study ESUS-classification does not include results of 72 h Holter-monitoring.

Strokes with vascular cause were discriminated according to the TOAST criteria [15]. ESUS was defined as proposed by the Cryptogenic Stroke/ESUS International Working Group [2]. Clinical work-up of all participants is provided in the supplement.

### Echocardiography

All participants were subjected to transthoracic echocardiography and data were collected prospectively according to the recommendations of the European Association of Cardiovascular Imaging [16, 17]. Detailed echocardiographic examination is described in the supplement.

Longitudinal strain- and strain rate analyses of the LA were performed offline (QLab 7.0, Philips Medical Systems, USA) using zoom mode images of the LA in four and two chamber views. The interval between the onset of P-wave in lead II of the ECG on echocardiographic images and the peak A`-wave of the lateral mitral valve (MV) annulus in tissue Doppler imaging was defined as PA-TDI lateral (lPA-TDI) [18, 19]. Furthermore, we measured this parameter at the septal MV annulus and defined the parameter as PA-TDI septal (sPA-TDI). LAVI was determined from biplane LA-measurements of images obtained in the 4- and 2-chamber views and LAVI/a` was ascertained as previously reported [10, 12].

### ECG

AF was determined on the basis of ECG findings. All participants had a 12-channel surface ECG recorded after inclusion in this study. Immediately after echocardiography, participants in the stroke collective were monitored with two-channel (five-lead) Holter-ECG-monitoring (GE Healthcare SEER™ 1000, Great Britain) scheduled for 72 h. Detailed ECG examination and work-up is described in the supplement.

### Statistical analysis

Nominally scaled parameters are given as *n* (%). Metric scaled variables are expressed as mean ± standard deviation (SD) for quantification of normally distributed variables, or median and interquartile ranges (IQR) in the tables for non-normally distributed variables.

Figures were created using GraphPad Prism 6.0 (GraphPad Software, Inc., La Jolla, CA). Data were analyzed with SPSS Statistics 25 (IBM SPSS Statistics 25). A two-sided *P* value of < 0.05 was considered statistically significant. Detailed statistical analysis is presented in the supplement.

## Results

### Baseline

**Fig. 1:**
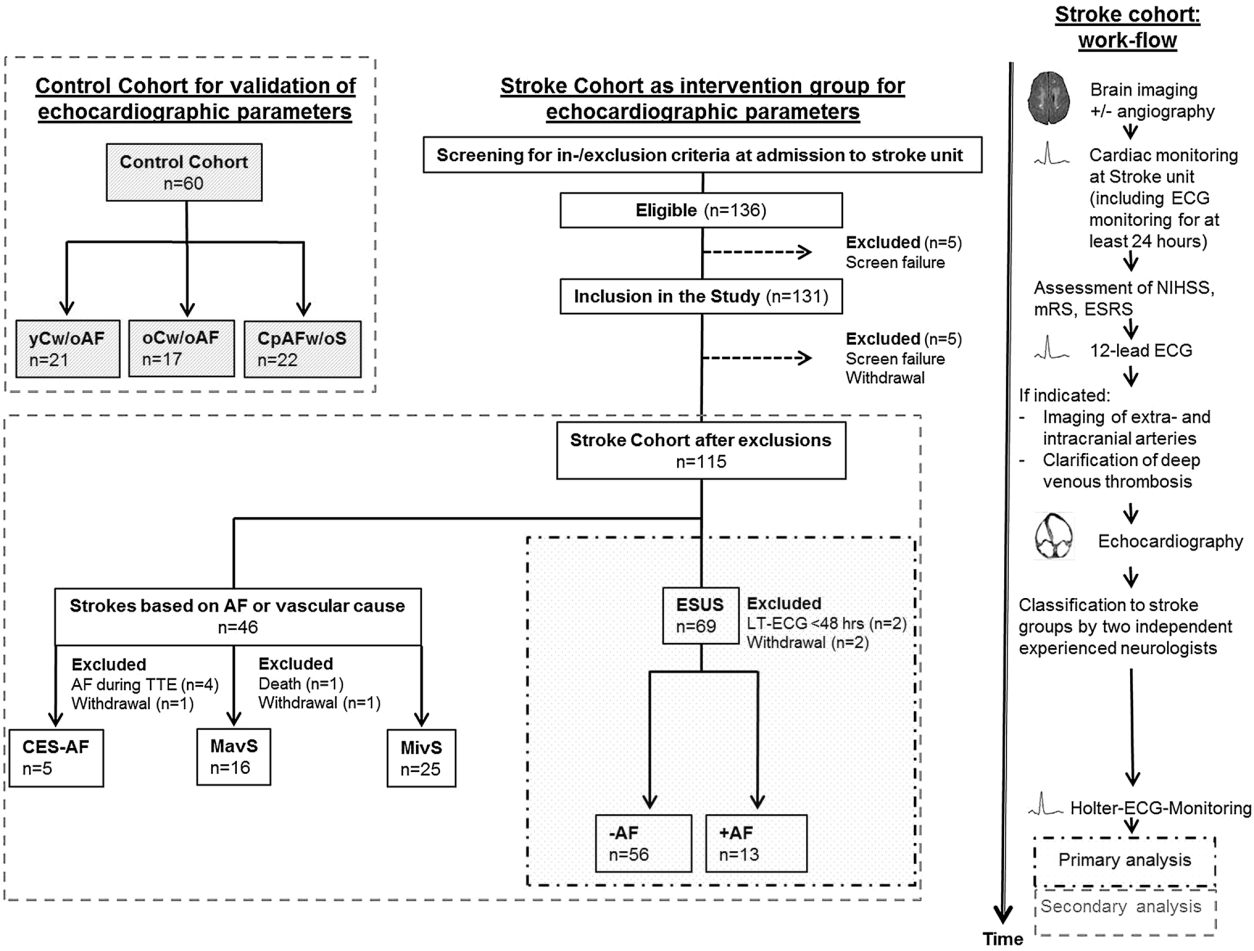
Study Enrollment. *AF* atrial fibrillation, *yCw/oAF* healthy, young volunteers, *oCw/oAF* healthy volunteers of old age without documented AF, *CpAFw/oS* participants with documented chronic paroxysmal AF without acute stroke, *ESUS* participants with embolic stroke of unknown source, *CES-AF* cardio-embolic stroke based on chronic paroxysmal AF, *MavS* stroke depend on macro-vascular cause, *MivS* stroke depend on micro-vascular cause

From 136 eligible stroke patients identified between 1st August 2016 and 30th April 2017 115 patients were included in this study after exclusion of 21 patients as described in Fig. 1 (stroke cohort). After completion of diagnostic stroke work-up stroke patients were classified into the following groups: ESUS (*n* = 69), CES-AF (*n* = 5), MavS (*n* = 16), MivS (*n* = 25). Moreover, participants serving as controls were included as follows: yCw/oAF (*n* = 21), CpAFw/oS (*n* = 22), oCw/oAF (*n* = 17).

Baseline characteristics of study participants are summarized in Table 1 and supplementary Table 1. Participants in the young control group (yCw/oAF) were significantly younger and had less preexisting risk factors/diseases in comparison to other groups. Risk stratification as well as stroke severity assessed with the Essen Stroke Risk Score (ESRS), National Institutes of Health Stroke Scale(NIHSS)and the modified Rankin Scale (mRS)were not significantly different between stroke groups.

**Table 1:** Baseline characteristics at inclusion

Parameter	yCw/oAF	CpAF w/oS	oCw/oAF	ESUS		CES-AF	MavS	MivS
	*n* = 21	*n* = 22	*n* = 17	− AF *n* = 56	+ AF *n* = 13	*n* = 5	*n* = 16	*n* = 25
Age (years)	29.8 ± 5.8	70.1 ± 10 ***	65.7 ± 10.7***	65 ± 13.7***	75 ± 9.7 ***	75 [70-86]***	70.7 ± 7.4***	68.3 ± 11.7***
Sex: female	5 (23.8%)	8 (36.4%)	6 (35.3%)	19 (33.9%)	5 (38.5%)	3 (60%)	4 (25%)	9 (32%)
Pre-existing conditions								
Hypertension	0	18 (81.8%)***	10(58.8%)***||	32 (57.1%)***	13 (100%)***	4 (80%)***	13(81.3%)***	21 (84%)***
Diabetes	0	3 (13.6%)	0§|##	11 (19.6%)*	4 (30.8%)**	1 (20%)*	1 (6.3%)	8 (32%)**
Stroke	0	2 (9.1%)*	2 (11.8%)*	9 (16.1%)*	3 (23.1%)*	0	7(43.8%)***§	9(36%)**§
CAD	0	8 (36.4%)**	2 (11.8%)	3(5.4%)†††	3(23.1%)*§	1(20%)*	4(25%)*§	4 (16%)
CHADS_2_	0 [0–0]	1.5 [1, 2] ***	1 [0–2]	1 [0–2]***	2 [1–3.5]***	4 [2.5–4]***	3.5 [2.25–4]***§§	2 [1–3] ***
0	21 (100%)	2 (9.1%)	6 (35.3%)	14 (25%)	0	0	0	2 (8%)
1		9(40.9%)	6 (35.3%)	20 (35.7%)	4 (30.7%)	0	2 (12.5%)	6 (24%)
2		7 (31.8%)	3 (17.6%)	12 (21.4%)	5 (38.5%)	1 (20%)	2 (12.5%)	8 (32%)
3		2 (9.1%)	1 (5.9%)	5 (8.9%)	1 (7.7%)	1 (20%)	4 (25%)	5 (20%)
4		0	1 (5.9%)	5 (8.9%)	1 (7.7%)	3 (60%)	5 (31.3%)	1 (4%)
5		2 (9.1%)	0	0	2 (15.4%)	0	3 (18.8%)	3 (12%)
Holter-ECG-Duration [h]			60 [24-72]	72 [60-72]	72 [67-72]		72 [48-72]	72 [36-72]
AF-Detection		22 (100%)	0†††‡‡‡|||	0†††‡‡‡|||	13 (100%)	5(100%)	0†††‡‡‡|||	0†††‡‡‡|||
P-wave duration [ms]	117 [112–119]	122 [109–128]§§	107 [93–115]	101 [93–112]**	105 [97–115]	114 [92–129]	97 [91–112]*†	106 [92–116]
PR-interval [ms]	155 [135–178]	176 [154–192]	162 [139–185]	157 [140–179]	176 [148-214]	194 [158–214]	167 [148–191]	161 [142–173]

*CAD* coronary artery disease, Variables are expressed as mean ± SD, median [IQR] or n (% of total number). CHADS_2_-Score was determined on medical history before acute stroke event at baseline

*p < 0.05 vs yCw/oAF **p < 0.01 vs yCw/oAF; ***p < 0.001 vs yCw/oAF

†*p* < 0.05 vs CpAFw/oS, ††*p* < 0.01 vs CpAFw/oS, †††*p* < 0.001 vs CpAFw/oS

‡*p* < 0.05 vs CES-AF, ‡‡‡*p* < 0.001 vs CES-AF

§*p* < 0.05 vs ESUS –AF, §§*p* < 0.01 vs ESUS-AF, §§§*p* < 0.001 vs ESUS-AF

|*p* < 0.05 vs ESUS +AF, ||*p* < 0.01 vs ESUS + AF, |||*p* < 0.001 vs ESUS + AF

#*p* < 0.05 vs MivS, ##*p* < 0.01 vs MivS,###*p* < 0.001 vs MiVs

### Holter-ECG-monitoring and 12-lead ECG

In stroke patients, the median time from admission to the emergency department to start of Holter-ECG-Monitoring was 48 (35.5–84.5) hours. The examination was started within 24 h after providing written consent. Two patients of the ESUS group had to be excluded because of a Holter-ECG-monitoring duration below 48 h. The detection rate of subclinical AF in the ESUS-group was 18.8%. Overall, we detected 24 episodes of AF in the ESUS-group with a median of 1 AF [1–3] episode per patient, a median duration of 58 [35–178] seconds and a minimum of 30 s. AF was not detected in the remaining stroke subgroups. Characteristics of Holter-ECG-monitoring and 12-lead ECG are also provided in Table 1. P-wave duration and PR-interval obtained in 12-lead ECGs were significantly altered in comparison of patients with AF and patients without AF. In univariate regression analysis of the complete cohort P-wave duration (*p* = 0.05) and PR-interval (*p* < 0.05) were predictors of AF (supplementary Table 3). Multivariate analysis showed that P-wave duration and PR-interval were not independently associated with AF in this study.

### Echocardiographic parameters

**Fig. 2:**
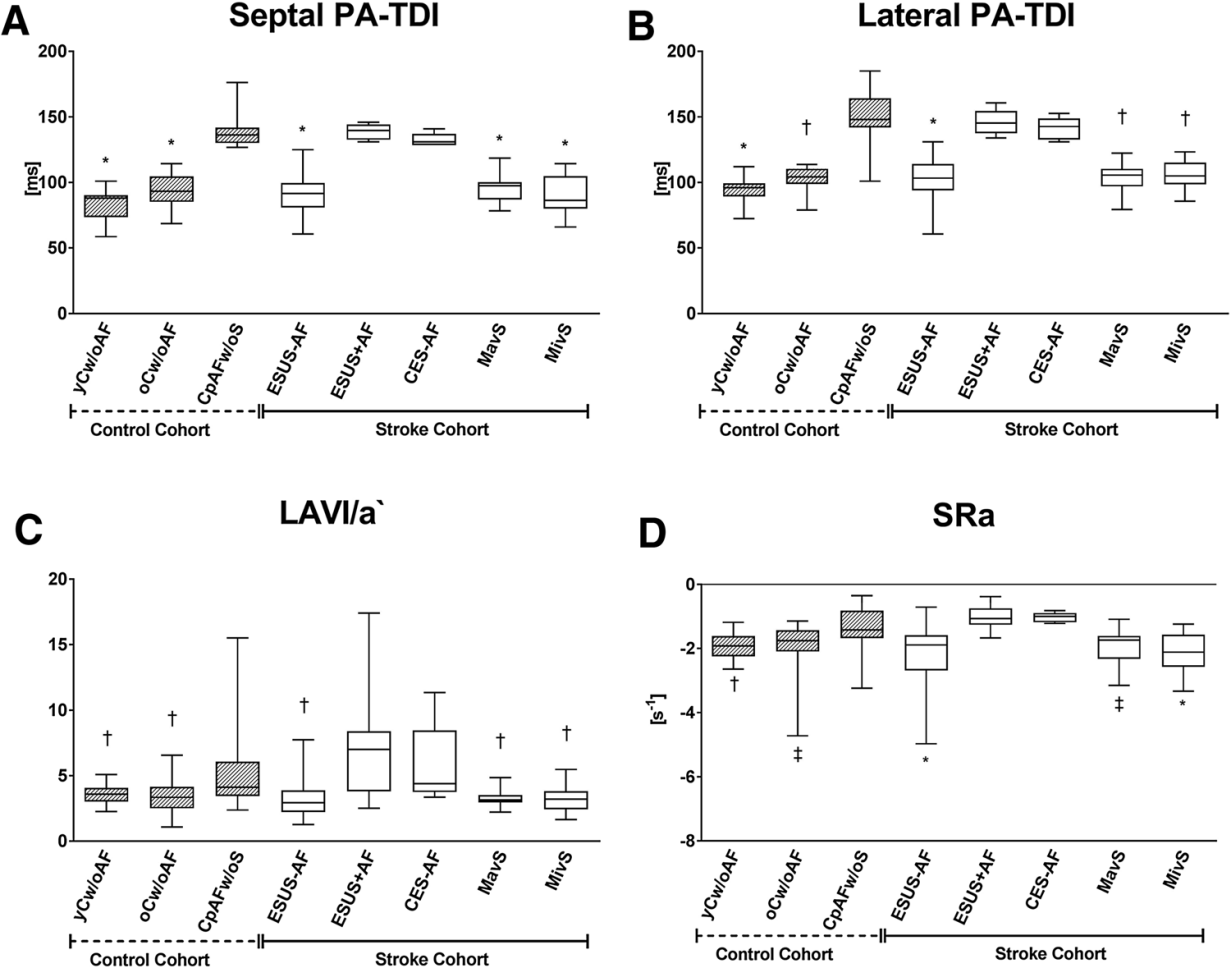
Echocardiographic parameters indicating left atrial remodeling are significantly altered in study populations with AF. **a** septal PA-TDI, **b** lateral PA-TDI, **c** LAVI/a`, **d** SRa. *p < 0.05 vs CpAFw/oS/ESUS + AF/CES-AF, †*p* < 0.05 vs. CpAFw/oS/ESUS + AF, ‡*p* < 0.05 vs ESUS + AF, §*p* < 0.05 MavS/MivS

**Fig. 3:**
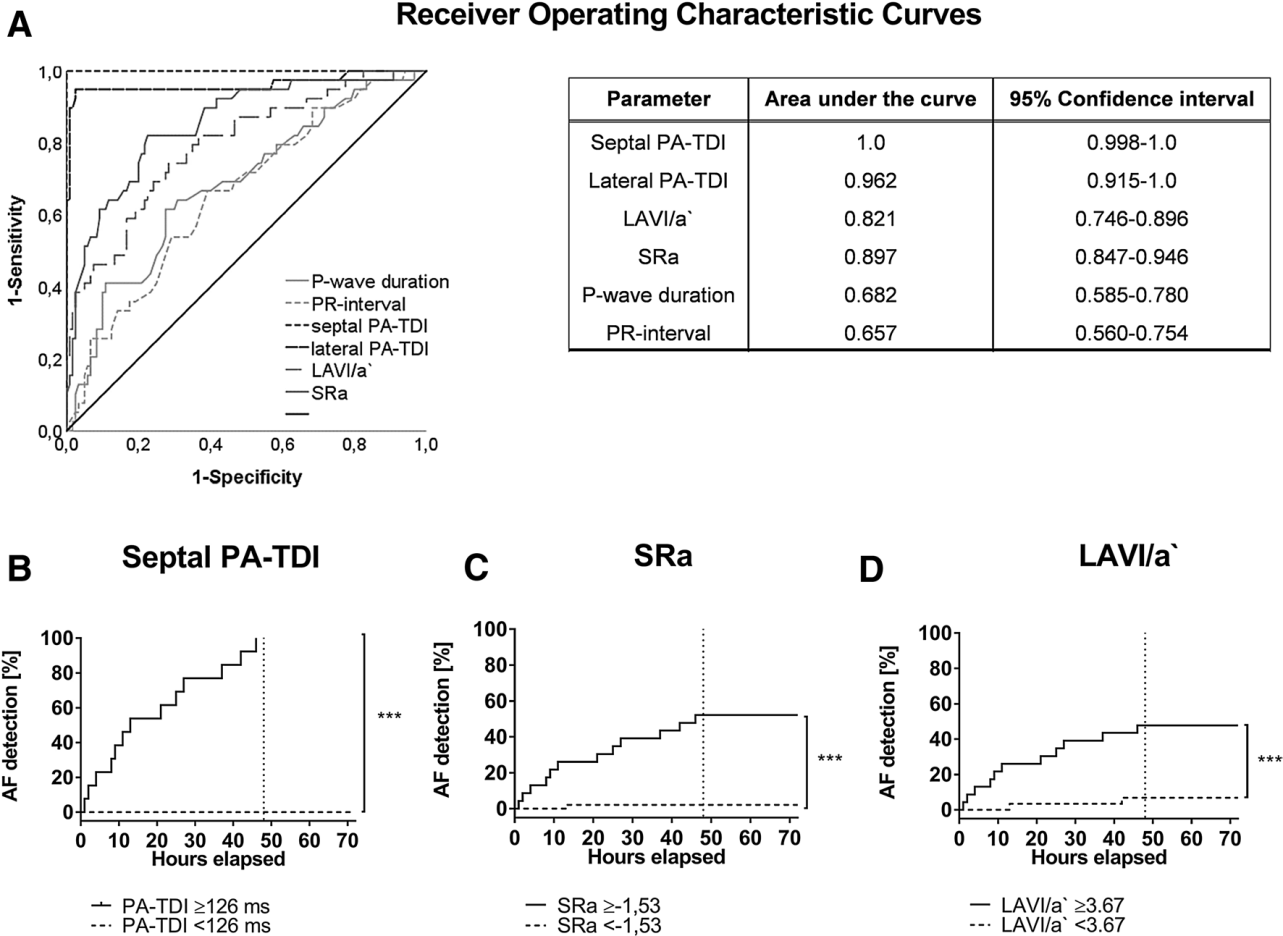
sPA-TDI interval, LAVI/a`andSRa predict subclinical AF in ESUS patients. **a** Receiver operating characteristic curves, **b** sPA-TDI cut-off, **c** SRa cut-off, **d** LAVI/a` cut-off. ***p < 0.001

Parameters of echocardiography are summarized in supplementary Table 2. There was no statistical significant difference for left ventricular ejection fraction as well as left and right ventricular diameters between patients with and without AF. Parameters of LV diastolic dysfunction (E`, E/A) were significantly different between young volunteers (yCw/oAF) and participants of the other groups. In patients with AF all parameters of LA size (LAVI) and function (LA-GLS, SRa, SRs, SRe, LAVI/a`) as well as septal and lateral PA-TDI differed in comparison to participants without AF, whereas there was no difference between the groups with preexisting or subclinical AF (Fig. 2, supplementary Table 2, supplementary Fig. 1).An atrial asynchrony represented by |ΔPA-TDI| was not significantly altered in comparison between the groups with or without AF (supplementary Table 2, supplementary Fig. 1). Univariate regression analysis showed global longitudinal strain (GLS), CHADS_2_, CHA_2_DS_2_-VASc, septal and lateral PA-TDI as predictors of AF in the complete cohort (supplementary Table 3). However, multivariate analyses highlighted sPA-TDI (*p* < 0.001), LA-VI/a` (*p* = 0.02), SRa (*p* = 0.05) and CHADS_2_ (*p* = 0.02) as independently associated with the presence of AF in the complete cohort (Table 2).CHADS_2_-VASc (*p* = 0.85), LAVI (*p* = 0.68), hypertension (*p* = 0.4), SVT (*p* = 0.3), CAD (*p* = 0.69) and lPA-TDI (*p* = 0.85) missed statistical significance. Receiver operating characteristic curve (ROC) analysis (Fig. 3a) and subsequent analysis of Youden`s index determined discriminators for AF detection as follows: sPA-TDI = 126 ms (sensitivity 99.9%, specificity 99.9%), LAVI/a` = 3.67 (sensitivity 78.1%, specificity 66.2%), SRa = − 1.53(sensitivity 87.2%, specificity 78.8%) (Fig. 3b–d). In addition, multivariate regressions analysis within the stroke cohort and the ESUS group confirmed only sPA-TDI as an independent predictor of subclinical AF (Table 2). In Kaplan–Meier curves sPA-TDI seems to be an incremental predictive parameter for subclinical AF: In all ESUS patients with a sPA-TDI ≥  26 ms but in none of the patients with a sPA-TDI < 126 ms AF was detected within 48 h of Holter-ECG-Monitoring (Fig. 3b).

**Table 2:** Predictors of AF in multivariate regressions analysis

Parameter	Complete Cohort (*n* = 175)		Stroke Cohort (*n* = 115)		ESUS (*n* = 69)	
	Multivariate regression analysis		Multivariate regression analysis		Multivariate Cox regression analysis	
	HR (95% CI)	*p* value	HR (95% CI)	*p* value	HR (95% CI)	*p* value
CHADS_2_-Score	2.0 (1.14–3.54)	0.02			1.02 (0.50–2.07)	0.961
PA-TDI septal	1.06 (1.04–1.08)	<0.001	1.18 (1.02–1.35)	0.024	1.10 (1.04–1.17)	0.001
LAVI/a`	0.85 (0.74–0.97)	0.02	0.82 (0.57–1.17)	0.27	0.89 (0.69–1.15)	0.36
SRa	2.35 (0.9–5.5)	0.05*	4.6 (0.19–107.8)	0.34	4.69 (0.26–84.0)	0.29

*CI* confidence interval, *HR*  hazard ratio

## Discussion

In the present prospective study, the echocardiographic parameter sPA-TDI strongly predicts the presence of subclinical AF in patients hospitalized with ESUS. Recent studies reported that in about 25% of ESUS patients subclinical AF can be detected by prolonged cardiac rhythm monitoring (implantable loop recorders) [6, 20]. However, prolonged cardiac rhythm monitoring employing extended Holter-ECG-monitoring and especially implantable loop recorders for detection of subclinical AF requires significant diagnostic resources. Especially patients with stroke and AF are known to benefit from therapeutic anticoagulation to prevent recurrent strokes. Current randomized trials investigated whether oral anticoagulation may be superior to platelet aggregation inhibition for secondary stroke prevention in ESUS patients [21–23]. However, the NAVIGATE ESUS trial has been recently stopped due to a negative result in the application of this approach including an increased risk of bleeding [22]. Hence, predictors of subclinical AF in ESUS might be important for risk-based decision making.

LA remodeling and in particular fibrosis displayed by late gadolinium enhancement (MRI), represents an important LA arrhythmic substrate [7] and LA fibrosis detected by MRI is strongly associated with the occurrence of stroke [7]. In addition, parameters of LA function and remodeling determined by echocardiography correlate with left atrial structural remodeling and function determined by MRI [9]. In line, LA enlargement following atrial structural remodeling is increased in patients with AF and predicts the occurrence of AF [24, 25]. Overall, the value of non-invasive evaluation of LA function by echocardiography has been increasingly demonstrated. Toh et al. [12] displayed that the ratio of indexed LA volume and mitral annulus velocity during atrial contraction (LAVI/a`), reflecting atrial pump function, facilitates diagnosis of prior AF. For AF prediction LAVI/a` cut-off values of 2.3 and 2.82 have previously been reported [10, 12]. However, in our study we defined a LAVI/a` cut-off value of > 3.67 (sensitivity 78.1%; specificity 66.2%) as independent predictor of AF (AUC = 0.798). P-wave duration and PR-interval obtained from resting 12-lead ECG have shown to be associated with myocardial structural remodeling and AF [26–28]. P-wave duration and PR-interval were not independently associated with AF in the present study.

LA receives blood via the pulmonary vein during LV systole and subsequently conduits blood after mitral valve opening to the LV during early diastole followed by active LA contraction and filling of the LV in late diastole [29]. Regional LA function and deformation representing this process can be displayed by strain and strain rate analysis with speckle tracking echocardiography [29]. In addition, strain and strain rate exhibit low inter- and intraobserver variability, are independent of tethering effects and were suggested for assessing myocardial function [30, 31]. Parameters of speckle tracking echocardiography are impaired with increased LA fibrosis [9] and alterations in strain rate analysis are independent of LA enlargement [32] but predictive for successful ablation of AF during follow-up [33]. In this study, all parameters of 2D speckle tracking echocardiography determined during sinus rhythm representing LA function were significantly decreased in groups with AF. Furthermore, all parameters were univariable predictors of paroxysmal AF. Of these SRa, representing the contractile phase of the LA, is an independent predictor for AF occurrence for all patients.

In our study the PA-TDI determined at the septal mitral valve ring was even superior to the lateral derived PA-TDI in predicting AF. One reason for the observed superiority of septal PA-TDI compared to (lateral) PA-TDI might be that measurements of septal compared to lateral TDI parameters have been described to be technically easier, less affected and, therefore, showing better diagnostic utility [34]. Multivariate analysis depicted sPA-TDI as the most powerful echocardiographic parameter (AUC = 1) predicting AF in our study. Subsequently, we defined an optimal sPA-TDI cut-off point of 126 ms (sensitivity 99.9%; specificity 99.9%) for the whole cohort. In the overall stroke collective as well as in ESUS patients sPA-TDI was the only independent predictor of AF.

To the best of our knowledge, no other study compared the value of PA-TDI, LAVI/a` and strain rate parameters for the prediction of subclinical AF in a detailed stroke collective and in particular ESUS. Furthermore, we described for the first time the parameter septal PA-TDI, which is superior to lateral PA-TDI in predicting AF in this study. Of note, septal PA-TDI can be easily determined during regular transthoracic echocardiography and does not require special echocardiography software and significant additional analytical time like it is needed for speckle tracking/strain analysis. Therefore, implementation into regular stroke work-up is feasible.

In this study, patients received telemetric Holter monitoring during Stroke-unit stay and immediately after echocardiography participants in the stroke cohort were monitored with a two-channel Holter-ECG. By this, subclinical AF was detected in 19% of patients with ESUS. Importantly, in all of these patients sPA-TDI was higher than the highly predictive sPA-TDI cut-off value for AF occurrence.

Recent studies indicate rather an extension of ECG monitoring [5] beyond the recommended 72 h by current guidelines of the European Society of Cardiology [36, 37] than reducing it to less. Current US guidelines even recommend continuous ECG monitoring over a time period of 30 days after cryptogenic stroke [35]. sPA-TDI may provide important additive information to increase the sensitivity to detect AF. Therefore, sPA-TDI may serve a powerful tool to decrease time- and resource-consuming extended ECG monitoring, e.g. repetitive Holter-ECG also affecting patient compliance. In addition, sPA-TDI may specifically help to identify high-risk patients which particularly benefit from extended monitoring by implanted loop recorders and avoid unnecessary implantation of these devices in low-risk patients.

Overall, our results suggest that echocardiographic parameters of adverse LA remodeling and dysfunction, especially sPA-TDI, may provide additive risk-stratified information to reduce duration of ECG monitoring after stroke.

The following aspects may limit our findings: (1) This was a single center pilot study. Management in stroke so as echocardiography is expertise-dependent. (2) In previous studies, significant differences in assessment of neurological severity in relation to the underlying cause of stroke are reported. In particular, patients with cardioembolic strokes had the most severe neurological restrictions [38, 39]. In respect of the exclusion criteria patients who gave written consent were included in this study. Hence, physical restricted patients were excluded. Therefore, in this study a difference in neurological impairment cannot be described as assessed by ESRS, NIHSS, and mRS. Furthermore, this exclusion criterion in combination with the inclusion criterion of sinus rhythm might have contributed to a limited number of patients included with cardioembolic stroke due to atrial fibrillation. (3) Due to the mental status of the patients and the goal of early neurological rehabilitation in these patients duration of Holter-ECG-monitoring differs in the stroke cohort.

Based on our data future studies should validate the predictive value of the identified echocardiographic parameters for AF employing continuous rhythm monitoring in an external population.

## Summary/conclusions

This study supported the remarkable potential of echocardiographic parameters indicating LA dysfunction and remodeling in predicting subclinical AF in patients with ESUS. In particular, the echocardiographic parameter sPA-TDI seems to be a very strong predictor of subclinical AF in patients hospitalized for ESUS and might support risk-stratified clinical decision making in these patients, which has to be investigated in further studies.

## Electronic supplementary material

Below is the link to the electronic supplementary material.

Supplementary material 1 (JPEG 276 kb)

Supplementary material 2 (DOCX 22 kb)

Supplementary material 3 (DOCX 43 kb)
