# Supplementary figures and images for: Septal total atrial conduction time for prediction of atrial fibrillation in embolic stroke of unknown source: a pilot study
Source: Clin Res Cardiol. 2019 Jun 24;109(2):205–14. doi: 10.1007/s00392-019-01501-2 (PMC6989646; doi:10.1007/s00392-019-01501-2)

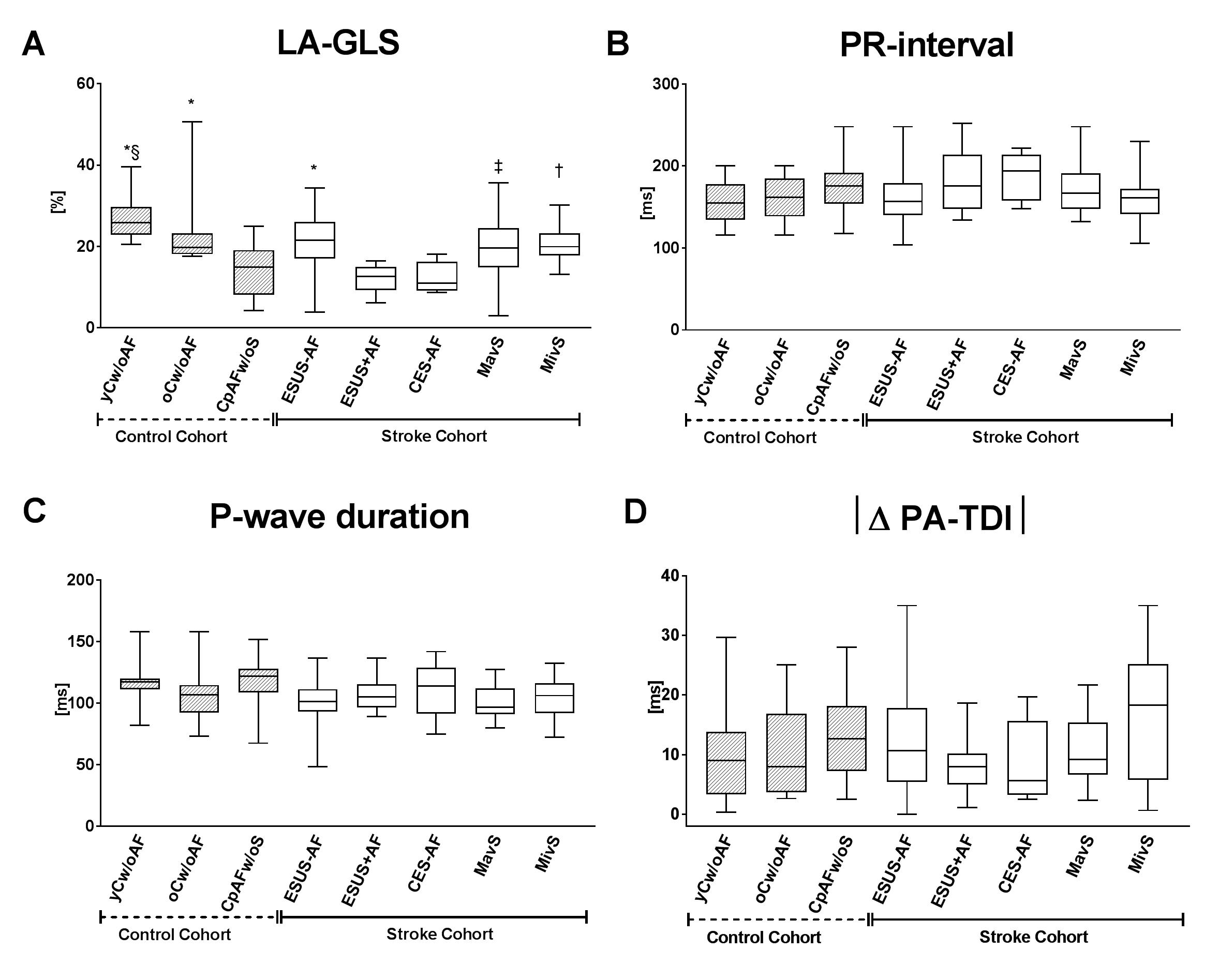

Supplement: Supplementary file 1 — Supplementary material 1 (JPEG 276 kb) [file 392_2019_1501_MOESM1_ESM.jpg]
