# Supplementary material for: Septal total atrial conduction time for prediction of atrial fibrillation in embolic stroke of unknown source: a pilot study
Source: Clin Res Cardiol. 2019 Jun 24;109(2):205–14. doi: 10.1007/s00392-019-01501-2 (PMC6989646; doi:10.1007/s00392-019-01501-2)
Supplement: Supplementary file 2 — Supplementary material 2 (DOCX 22 kb) [file 392_2019_1501_MOESM2_ESM.docx]

**ONLINE SUPPLEMENT**

**Septal Total Atrial Conduction Time for prediction of Atrial Fibrillation in Embolic Stroke of Unknown Source - a Pilot Study**

Jan-ThorbenSieweke, MD^1*^; Saskia Biber^1*^; Karin Weissenborn, MD^2^; Peter U. Heuschmann, MD^3^; Muharrem Akin,MD^1^; Florian Zauner, MD^1^; Maria M. Gabriel, MD^2^; Ramona Schuppner, MD^2^; Dominik Berliner, MD^1^ ; Johann Bauersachs, MD^1^; Gerrit M. Grosse, MD^2‡^; Udo Bavendiek, MD^1‡§^

^*^ Authors contributed equally as first authors; ^‡^ Authors contributed equally as senior authors

^1^ Department of Cardiology and Angiology, ^2^Department of Neurology

^1+2^ Hannover Medical School, Hannover, Germany

^3^ Insitute of Clinical Epidemiology and Biometry, University Würzburg; Comprehensive Heart Failure Center,

University of Würzburg; Clinical Trial Center, University Hospital Würzburg, Würzburg, Germany

**Supplemental Methods**

**Inclusion and Exclusion criteria:**

Inclusion criteria for stroke patients was an acute focal neurological symptom with accompanying evidence of cerebral infarction in diffusion weighted (DWI) magnetic resonance imaging (MRI) or in cranial computer tomography (CT) with CT-angiography. Exclusion criteria were: age under 18 years, AF during echocardiographic examination (as parameters for LA remodeling cannot be determined in presence of AF), acute myocardial infarction, history of myocardial infarction, severe mitral valve stenosis or regurgitation, history of aortic or mitral valve replacement, ablation of supraventricular tachycardia, hemorrhagic stroke and patients, who could not provide informed consent. Stroke patients with competing or rare etiologies like e.g. vasculitis, endocarditis, dissection of the brain supplying arteries, or evidence of deep vein thrombosis in combination with patent Foramen ovale (PFO) were excluded. Furthermore, ESUS-patients with Holter-ECG-monitoring <48 hours were not considered.

**Clinical work-up:**

All participants underwent 12-lead electrocardiogram (ECG) and detailed transthoracic echocardiography. Clinical work-up was done as follows: 1.) Control cohort: Participants in the control cohort had no acute stroke. Holter-ECG-monitoring was not performed in yCw/oAF. Participants were included in the oCw/oAF group, if there was no history of AF and/or no documented AF in a 24h-LT-ECG. In contrast, patients with known paroxysmal AF hospitalized for catheter ablation of AF were included in the CpAFw/oS group. 2.) Stroke cohort: All patients with acute ischemic stroke were treated at a stroke unit certified by the German Stroke Society. Clinical workup of stroke patients included best medical care, neurological examination along with assessment of the National Institutes of Health Stroke Scale (NIHSS), the modified Rankin Scale (mRS), the Essen Stroke Risk Score (ESRS), monitoring of vital parameters during Stroke Unit stay for at least 48h and an additional Holter-ECG-Monitoring scheduled for 72h beginning after transthoracic echocardiography. CHADS_2_- and CHA_2_DS_2_-VASc Score were determined based on medical history present before acute stroke event at baseline. Echocardiography and Holter-ECG-Monitoring was applied by trained study personnel.

**Echocardiographic examination:**

Transthoracic echocardiography was performed in the left lateral decubitus with a commercially available cardiology-ultrasound machine (Epiq7, Philips Medical Systems, USA) equipped with a 5-1 MHz transducer. Complete echocardiographic examination was performed during brief breath hold. Parameters of diastolic function were assessed by pulsed wave Doppler imaging at the tips of the mitral leaflets (E- and A-wave of mitral valve inflow) and by tissue Doppler imaging at the level of the septal and lateral mitral valve (MV) annulus, respectively. Three to five consecutive cardiac cycles of each view were acquired during passive end-expiration and stored digitally as raw data for further offline analysis. 2D speckle-tracking of the LA was performed by tracing the endocardial border with an automatic tracing function. In case of deviation of this function, tracing was manually corrected. PA-TDI intervals were acquired three times per patient and the average value was calculated. Moreover, strain rate was evaluated by measuring the peak positive strain rate (SRs) during LV systole, the first negative peak strain rate (SRe) during early diastole, and the second negative peak strain rate (SRa) during atrial contraction [1].In intention to detect an atrial asynchrony we defined the parameter | ΔPA-TDI| [= |(lateral PA-TDI-septal PA-TDI)|].

**ECG examination and work-up:**

One investigator blinded to clinical and echocardiographic results analyzed 12-lead ECGs. P-wave duration and PR-interval was automatically measured by the ECG-device or was compiled using a median beat in respect of information from all P-waves documented in all 12 leads. 12-lead ECGs with AF, second- and third-degree of AV-block or delta waves were not considered for the analysis. Holter-ECG-monitoring was not performed in the young control group (yCw/oAF) without a history of AF. Participants were included in the oCw/oAF group, if there was no history of AF and/or no documented AF in a 24h-LT-ECG (available in 12 of 17 patients). All ECG recordings were assessed offline using analysis software (CardioDay; getemedMedizin- und Informationstechnik) by two independent professionals, who were blinded to echocardiographic and clinical data and taking into account the diagnostic criteria of current guidelines on atrial fibrillation[2].

**Statistical analysis:**

Normality and variance homogeneity were checked by Shapiro-Wilk and D`Agostino Pearson test. Statistical analysis was performed with ANOVA and Mann-Whitney test as nonparametric test followed by Bonferroni test or Dunn`s test for multiple comparisons, respectively. Chi-square test was applied to compare nominally scaled patient characteristics. Univariate regression analysis was performed including all variables potentially associated with AF (p<0.05). Subsequently predictors of AF were determined using a stepwise multivariate regression analysis with variables, which significantly linked to AF in univariate analysis (p<0.05). Prediction of AF in the whole cohort and the stroke cohort were tested with logistic regression analysis. Prediction in ESUS patients during Holter-ECG-Monitoring was tested with Cox regression proportionality analysis. Results from the regression analyses are presented as hazard ratios (HRs) with 95% confidence intervals (CIs). The discriminative ability of the risk prediction model was assessed by the area under the receiver operating characteristic (ROC) curve. Youden`s index was determined to ascertain cut-off values of the variables independently associated with AF. Cumulative new onset of AF for the variables was estimated by Kaplan-Meier method and cut-off values were compared by the log-rank test.

**Suppl. Figure 1: Parameters potentially associated with AF in study populations**

**A:** LA-GLS,**B:** PR-interval,**C:**P-wave duration,**D:** |ΔPA-TDI|

*p<0.05 vs CpAFw/oS/ESUS+AF/CES-AF, †p<0.05 vs.CpAFw/oS/ ESUS+AF, ‡p<0.05 vs ESUS+AF, §p<0.05 MavS/MivS

**Supplemental References**

1. Sun JP, Yang Y, Guo R, Wang D, Lee AP, Wang XY, Lam YY, Fang F, Yang XS, Yu CM (2013) Left atrial regional phasic strain, strain rate and velocity by speckle-tracking echocardiography: normal values and effects of aging in a large group of normal subjects. Int J Cardiol 168:3473-3479

2. Kirchhof P, Benussi S, Kotecha D, Ahlsson A, Atar D, Casadei B, Castella M, Diener HC, Heidbuchel H, Hendriks J, Hindricks G, Manolis AS, Oldgren J, Popescu BA, Schotten U, Van Putte B, Vardas P, Agewall S, Camm J, Baron Esquivias G, Budts W, Carerj S, Casselman F, Coca A, De Caterina R, Deftereos S, Dobrev D, Ferro JM, Filippatos G, Fitzsimons D, Gorenek B, Guenoun M, Hohnloser SH, Kolh P, Lip GY, Manolis A, McMurray J, Ponikowski P, Rosenhek R, Ruschitzka F, Savelieva I, Sharma S, Suwalski P, Tamargo JL, Taylor CJ, Van Gelder IC, Voors AA, Windecker S, Zamorano JL, Zeppenfeld K (2016) 2016 ESC Guidelines for the management of atrial fibrillation developed in collaboration with EACTS. Europace 18:1609-1678
