# Supplementary material for: Septal total atrial conduction time for prediction of atrial fibrillation in embolic stroke of unknown source: a pilot study
Source: Clin Res Cardiol. 2019 Jun 24;109(2):205–14. doi: 10.1007/s00392-019-01501-2 (PMC6989646; doi:10.1007/s00392-019-01501-2)
Supplement: Supplementary file 3 — Supplementary material 3 (DOCX 43 kb) [file 392_2019_1501_MOESM3_ESM.docx]

**SUPPLEMENTAL MATERIAL**

**Septal Total Atrial Conduction Time for prediction of Atrial Fibrillation in Embolic Stroke of Unknown Source - a Pilot Study**

Jan-Thorben Sieweke, MD^1*^; Saskia Biber^1*^; Karin Weissenborn, MD^2^; Peter U. Heuschmann, MD^3^; Muharrem Akin,MD^1^; Florian Zauner, MD^1^; Maria M. Gabriel, MD^2^; Ramona Schuppner, MD^2^; Dominik Berliner, MD^1^ ; Johann Bauersachs, MD^1^; Gerrit M. Grosse, MD^2‡^; Udo Bavendiek, MD^1‡§^

^*^ Authors contributed equally as first authors; ^‡^ Authors contributed equally as senior authors

^1^ Department of Cardiology and Angiology, ^2^Department of Neurology

^1+2^ Hannover Medical School, Hannover, Germany

^3^ Insitute of Clinical Epidemiology and Biometry, University Würzburg; Comprehensive Heart Failure Center,

University of Würzburg; Clinical Trial Center, University Hospital Würzburg, Würzburg, Germany

§: To whom correspondence should be addressed: [bavendiek.udo@mh-hannover.de](mailto:bavendiek.udo@mh-hannover.de)

**Supplemental Tables**

**Supp. Table 1:** Baseline Characteristics at admission/ study inclusion

| **Parameter** | | **yCw/oAF** | **CpAF w/oS** | **oCw/oAF** | **ESUS** | | **CES-AF** | **MavS** | **MivS** |
| --- | --- | --- | --- | --- | --- | --- | --- | --- | --- |
|  |  | n=**21** | n=**22** | n=**17** | **-** AF  n= **56** | **+** AF  n= **13** | n=**5** | n=**16** | n=**25** |
| Height [cm] | | 181.7±7.8 | 170.8±9.4 ** | 172.3±9.2 | 173±8.4** | 169.1±9.8** | 168 [161-177] | 174.3±10.6 | 171.9±10** |
| Weight [kg] | | 80.6±11 | 79.9±13.7 | 79.6±12.3 | 79.5±13.3 | 79.5±16.5 | 65 [57.5-82] | 78.8±13.6 | 84±17 |
| In-hospital stay [days] | |  |  |  | 8 [6-10] | 7.9±2.5 | 7 [5-11.5] | 8 [5.3-13.5] | 7.6±3.3 |
| Stay in Stroke Unit [days] | |  |  |  | 3 [2.5-4] | 3 [2-3.5] | 2 [1.5-5] | 3 [2-6.8] | 3 [3-4] |
| Pre-existing conditions | |  |  |  |  |  |  |  |  |
|  | SVT | 0 †††‡‡‡ | 22 (100%) | 0 †††‡‡‡ | 0 †††‡‡‡ | 0†††‡‡‡ | 5 (100%) | 0†††‡‡‡ | 0†††‡‡‡ |
|  | Nicotine | 2 (9.5%) | 6 (27.3%) | 8 (47.1%) | 16 (28.6%) | 1 (7.7%) | 0 | 7(43.8%) | 7 (28%) |
|  | PAD | 0 | 3 (13.6%) | 2 (11.8%) | 2 (3.6%) | 1 (7.7%) | 0 | 0 | 3 (12%) |
| ESRS | | 0 [0-0] | 3 [2-4]*** | 2.2±1.4*** | 2 [1-3]*** | 3.3±1.5*** | 3 [2.5-3]* | 3±1.2*** | 3.1±1.7*** |
| NIHSS | | 0 [0-0] | 0 [0-0] | 0 [0-1]# | 2 [0.5-3.5] ***††† | 2 [1-4] ***††† | 3 [0.5-13] *† | 2.5 [1-3] ***††† | 3 [2-5] ***††† |
| mRS | | 0 [0-0] | 0 [0-0] | 0 [0-2]§\| | 2 [1-3] ***††† | 2.8±0.9***††† | 3 [1-4]*†† | 2 [1-2.8]*** ††† | 3 [2-4]***††† |
| CHA_2_DS_2_VASc | | 0 [0-0.5] | 3.5 [1-4.3]*** | 1 [1-3] | 2 [1-3.5]*** | 3 [2.5-5]*** | 4 [2.5-4]** | 3.5 [2.25-4]*** | 3 [2-4]*** |
|  | 0 | 16 (76.2%) | 1 (4.5%) | 2 (11.8%) | 6 (10.7%) | 0 | 0 | 0 | 1 (4%) |
|  | 1 | 5 (23.8%) | 5 (22.7%) | 8 (47.1%) | 15 (26.8%) | 2 (15.4%) | 0 | 2 (12.5%) | 3 (12%) |
|  | 2 | 0 | 1 (4.5%) | 3 (17.6%) | 11 (19.6%) | 1 (7.7%) | 1 (20%) | 2 (12.5%) | 5 (20%) |
|  | 3 | 0 | 4 (18.2%) | 0 | 10 (17.9%) | 4 (30.7%) | 1 (20%) | 4 (25%) | 5 (20%) |
|  | 4 | 0 | 6 (27.3%) | 3 (17.6%) | 7 (12.5%) | 2 (15.4%) | 3 (60%) | 5 (31.3%) | 6 (24%) |
|  | 5 | 0 | 3 (13.6%) | 1 (5.9%) | 5 (8.9%) | 3 (23.1%) | 0 | 3 (18.8%) | 1 (4%) |
|  | 6 | 0 | 0 | 0 | 2 (3.6%) | 1 (7.7%) | 0 | 0 | 2 (8%) |
|  | 7 | 0 | 2 (9.1%) | 0 | 0 | 0 | 0 | 0 | 1 (4%) |
|  | 8 | 0 | 0 | 0 | 0 | 0 | 0 | 0 | 0 |
|  | 9 | 0 | 0 | 0 | 0 | 0 | 0 | 0 | 1 (4%) |

AF- Atrial fibrillation, ESRS- Essen Stroke Risk Score, mRS- modified Rankin Scale, NIHSS- National institutes of Health Stroke Scale, PAD- peripheral artery disease, SVT- supraventricular tachycardia. CHA_2_DS_2_-VASc Score was determined on medical history before acute stroke event at baseline.

*p<0.05 vs yCw/oAF **p<0.01 vs yCw/oAF; ***p<0.001 vs yCw/oAF

†<p0.05 vs CpAFw/oS, ††p<0.01 vs CpAFw/oS, †††p<0.001 vs CpAFw/oS

‡p<0.05 vs CES-AF, ‡‡‡p<0.001 vs CES-AF

§p<0.05 vs ESUS –AF, §§p<0.01 vs ESUS-AF, §§§p<0.001 vs ESUS-AF

| p<0.05 vs ESUS +AF, || p<0.01 vs ESUS+AF, ||| p<0.001 vs ESUS+AF

#p<0.05 vs MivS, ##p<0.01 vs MivS,### p<0.001 vs MiVs

**Supp. Table 2: Characteristics of transthoracic echocardiography**

| **Parameter** | | **yCw/oAF** | **CpAF w/oS** | **oCw/oAF** | **ESUS** | | **CES-AF** | **MavS** | **MivS** |
| --- | --- | --- | --- | --- | --- | --- | --- | --- | --- |
|  | | n= **21** | n= **22** | n= **17** | **-** AF  n= **56** | **+** AF  n= **13** | n= **5** | n= **16** | n= **25** |
| LVEF [%] | | 62.4±3.7 | 59.5±6.4 | 57.8±6.2 | 61.5 [56.9-64.9] | 60.3 [49.9-64] | 55.7 [44.8-57.8] | 59 [55-63.7] | 58.8±4.5 |
| Diameter LV [cm] | | 5.2±0.5 | 5 [4.7-5.4] | 5±0.4 | 4.8±0.6 | 5±0.6 | 4.9 [4.6-5.4] | 4.8±0.6 | 4.8±0.7 |
| Diameter RV [cm] | | 3.9±0.5 | 3.5±0.6 | 3.6±0.7 | 3.6±0.5 | 3.9±0.6 | 3.4 [2.9-4.2] | 3.8±0.7 | 3.5 [2.9-3.9] |
| E`septal [cm/s] | | 10.8±1.7 | 6±1.7*** | 6.6±1.5** | 6 [4.6-6.9]*** | 5.8±1.8*** | 4.9 [3.2-5.7]*** | 5.6±1.8*** | 5.4±1.6*** |
| E`lateral [cm/s] | | 16.5±4 | 7.8±2.4*** | 9.4±2.7*** | 7.8±2.5*** | 7.4±2.2*** | 8.8 [4.8-10.8]*** | 7.7±3.6*** | 7.6±2.1*** |
| E/E`septal | | 7.9±1.8 | 9.9 [7.5-13.4] | 8.9 [8.1-11.7] | 10.6 [8.7-13.6]** | 8.9 [8.3-18.6] | 10.2 [7.8-18.5] | 10.2 [8.5-16.5]* | 12.1 [9-14.8] *** |
| E/E`lateral | | 5.2 [4.7-5.7] | 7.4 [6.3-10] ** | 7.1 [5.2-8.8] | 8.2 [6.5-11.4 ]*** | 9.2±4.1* | 7.5 [3.9-13.3] | 10 [5.6-12.9] ** | 8.3 [5.9-11.4] *** |
| A`septal [cm/s] | | 8.7±1.5 | 8.7±2.5 | 10.4±1.8 | 10.1±2.2 | 7.8±2.4 | 8.2 [7-10.8] | 9.7±1.6 | 9 [8.2-11.2] |
| A`lateral [cm/s] | | 9.6±1.5 | 10.7±3.6 | 11.8±1.9 | 11.8±2.7 | 7.4 [6.4-12.1] | 8.6 [6.9-10.4] | 11.3±3.2 | 10.8 [10.2-13] |
| MV E/A | | 1.7±0.5 | 0.8 [0.7-1.1] *** | 0.9±0.3  *** | 0.8 [0.7-1]*** | 0.7 [0.5-1.2] *** | 0.7 [0.5-0.8] *** | 0.8±0.2  *** | 0.8±0.2  *** |
| PA-TDI septal [ms] | | 84±12.2 | 136 [130-141.3] *** | 92.9±12.9  †††\|\|\| | 91.3±13.5  ††† | 139.1±5.6 ***§§§ | 131 [128.6-137] **§ | 95±10.2  †††\|\| | 90±13.7  †††‡\|\|\| |
| PA-TDI lateral [ms] | | 94.6±9.5 | 151±15.4  *** | 104.3 [98.8-110.4] †††\|\|\| | 103±14.5  ††† | 146.4±9.1  ***§§§ | 142.7 [132.7-148.8]***§ | 103.5±11.6  †††\|\|\| | 105.9±10.8  †††\|\|\| |
| \|ΔPA-TDI\| [ms] | | 9 [3.3-13.8] | 12.7 [7.3-18.2] | 8 [3.7-16.9] | 11 [5.4-17.8] | 8 [5-10.2] | 5.7 [3.3-15.7] | 9.2 [6.7-15.4] | 18.3 [5.8-25.2] |
| LAVI/a` | | 3.6±0.8 \|\|\| | 4.1 [3.4-5.6] | 3.3±1.3 †\|\| | 2.9 [2.2-3.9]†††\|\|\| | 7 [3.8-8.4] | 4.4 [3.8-8.5] | 3.1 [2.9-3.5]  †\|\|\| | 3.2±1 ††\|\|\| |
| LA-GLS [%] | | 26.5±4.7 | 14.5±5.7*** | 22.4±7.6††\|\|\|‡ | 21.5±6.8†††\|\|\|‡ | 12±3.3*** | 11[9.2-16.3]*** | 19.4±8*\| | 20.5±4.4*†\|\| |
| Strain rate (SR) analysis of LA | |  |  |  |  |  |  |  |  |
|  | SRs [s^-1^] | 1.9±0.4 | 1.1 [0.8-1.4]*** | 1.5 [1.3-1.8] † | 1.5 [1.2-1.9]\|\| | 1.1±0.3*** | 0.9 [0.8-1.6]* | 1.5±0.2 | 1.4±0.3* |
|  | SRe [s^-1^] | -2.6±0.6 | -1 [-1.4- -0.7]*** | -1.4±0.4** | -1.4±0.5***\| | -0.8 [-1- -0.6]*** | -0.7 [-1.8- - 0.6]** | -1.3±0.5*** | -1.2±0.4*** |
|  | SRa [s^-1^] | -1.9±0.4 | -1.4 [-1.7- -0.9] * | -1.8 [-2.1- -1.4] \| | -1.9[-2.7- -1.6] †††‡‡\|\|\| | -1±0.4** | -1 [-1.2- -0.9] | -1.9±0.4$$ | -2.1±0.6 ††\|\|\| |

AF- Atrial fibrillation, LA- left atrium, LAVI- left atrial volume index, LV- left ventricle, MV- mitral valve, RV- right ventricle

*p<0.05 vs yCw/oAF **p<0.01 vs yCw/oAF; ***p<0.001 vs yCw/oAF

†<p0.05 vs CpAFw/oS, ††p<0.01 vs CpAFw/oS, †††p<0.001 vs CpAFw/oS

‡p<0.05 vs CES-AF,‡‡‡p<0.001 vs CES-AF

§p<0.05 vs ESUS –AF, §§p<0.01 vs ESUS-AF, §§§p<0.001 vs ESUS-AF

| p<0.05 vs ESUS +AF, || p<0.01 vs ESUS+AF, ||| p<0.001 vs ESUS+AF

#p<0.05 vs MivS, ##p<0.01 vs MivS, ### p<0.001 vs MivS

**Supp. Table 3:** Univariate regression analysis to predict subclinical AF

| **Parameter** | **Complete Cohort**  (n=175) | | **Stroke Cohort**  (n=115) | | **ESUS**  (n=69) | |
| --- | --- | --- | --- | --- | --- | --- |
|  | **Univariate regression analysis** | | **Univariate regression analysis** | | **Univariate Cox regression analysis** | |
|  | HR (95%CI) | p-value | HR (95%CI) | p-value | HR (95%CI) | p-value |
| Hypertension | 11.30 (1.47-87.78) | 0.02 | 8.24 (1.1-64.70) | 0.04 | 6.76 (0.9-50.8) | 0.063 |
| History of CAD | 1.85 (0.56.6.15) | 0.32 | 2.26 (0.63-8.09 | 0.21 | 1.92 (0.63-5.84) | 0.25 |
| CHADS_2_-Score | 1.56 (1.14-2.12) | 0.005 | 1.38 (0.99-1.93) | 0.06 | 1.39 (1.00-1.93) | 0.05 |
| CHADS_2_-VASc | 1.33 (1.03-1.72) | 0.029 | 1.25 (0.94-1.67) | 0.13 | 1.33 (1.00-1.75) | 0.047 |
| PA-TDI septal | 1.09 (1.05-1.13) | 0.001 | 1.09 (1.05-1.13) | 0.001 | 1.11 (1.06-1.15) | 0.001 |
| PA-TDI lateral | 1.07 (1.04-1.09) | 0.001 | 1.07 (1.04-1.09) | 0.001 | 1.09 (1.04-1.12) | 0.001 |
| LAVI/a` | 1.54 (1.23-1.93) | 0.001 | 2.48 (1.64-3.74) | 0.001 | 1.19 (1.07-1.32) | 0.001 |
| LA-GLS | 0.79 (0.72-0.88) | 0.001 | 0.76 (0.66-0.86) | 0.001 | 0.84 (0.76-0.91) | 0.001 |
| SRs | 0.65 (0.01-0.29) | 0.065 | 0.037 (0.01-0.23) | 0.001 | 0.07 (0.02-0.37) | 0.001 |
| SRe | 7.52 (2.05-27.65) | 0.002 | 7.07 (1.79-27.80) | 0.005 | 4.92 (1.53-15.78) | 0.008 |
| SRa | 32.24 (7.32-141.9) | 0.001 | 157.6 (14.81-1676.85) | 0.001 | 5.76 (2.16-15.37) | 0.001 |
| P-wave duration | 1.032 (1.01-1.06) | 0.05 | 1.026 (0.99-1.06) | 0.133 | 1.02 (0.98-1.05) | 0.436 |
| PR-interval | 1.015 (1.004-1.026) | 0.019 | 1.014 (1.001-1.028) | 0.051 | 1.01 (0.99-1.03) | 0.077 |

CI- confidence interval, HR- hazard ratio, LA-GLS- left atrial global longitudinal strain
